# Adaptation mechanism of interlimb coordination in human split-belt treadmill walking through learning of foot contact timing: a robotics study

**DOI:** 10.1098/rsif.2015.0542

**Published:** 2015-09-06

**Authors:** Soichiro Fujiki, Shinya Aoi, Tetsuro Funato, Nozomi Tomita, Kei Senda, Kazuo Tsuchiya

**Affiliations:** 1 Department of Aeronautics and Astronautics, Graduate School of Engineering, Kyoto University, Kyoto daigaku-Katsura, Nishikyo-ku, Kyoto 615-8540, Japan; 2 Department Mechanical Engineering and Intelligent Systems, Graduate School of Informatics and Engineering, The University of Electro-Communications, 1-5-1 Choufugaoka, Choufu-shi, Tokyo 182-8585, Japan; 3 Department of Mathematics, Graduate School of Science, Kyoto University, Kitashirakawa-oiwakecho, Sakyo-ku, Kyoto 606-8502, Japan; 4 JST, CREST, 5 Sanbancho, Chiyoda-ku, Tokyo 102-0075, Japan

**Keywords:** split-belt treadmill walking, biped robot, learning, foot-contact timing, interlimb coordination, central pattern generator

## Abstract

Human walking behaviour adaptation strategies have previously been examined using split-belt treadmills, which have two parallel independently controlled belts. In such human split-belt treadmill walking, two types of adaptations have been identified: early and late. Early-type adaptations appear as rapid changes in interlimb and intralimb coordination activities when the belt speeds of the treadmill change between tied (same speed for both belts) and split-belt (different speeds for each belt) configurations. By contrast, late-type adaptations occur after the early-type adaptations as a gradual change and only involve interlimb coordination. Furthermore, interlimb coordination shows after-effects that are related to these adaptations. It has been suggested that these adaptations are governed primarily by the spinal cord and cerebellum, but the underlying mechanism remains unclear. Because various physiological findings suggest that foot contact timing is crucial to adaptive locomotion, this paper reports on the development of a two-layered control model for walking composed of spinal and cerebellar models, and on its use as the focus of our control model. The spinal model generates rhythmic motor commands using an oscillator network based on a central pattern generator and modulates the commands formulated in immediate response to foot contact, while the cerebellar model modifies motor commands through learning based on error information related to differences between the predicted and actual foot contact timings of each leg. We investigated adaptive behaviour and its mechanism by split-belt treadmill walking experiments using both computer simulations and an experimental bipedal robot. Our results showed that the robot exhibited rapid changes in interlimb and intralimb coordination that were similar to the early-type adaptations observed in humans. In addition, despite the lack of direct interlimb coordination control, gradual changes and after-effects in the interlimb coordination appeared in a manner that was similar to the late-type adaptations and after-effects observed in humans. The adaptation results of the robot were then evaluated in comparison with human split-belt treadmill walking, and the adaptation mechanism was clarified from a dynamic viewpoint.

## 1. Introduction

Human beings walk adaptively in various environments by generating appropriate motor commands in their neural systems. However, because the walking behaviour of humans also involves coordinating the movements of numerous joints, motor commands must create proper movement relationships between legs (interlimb coordination) and between the joints of each leg (intralimb coordination) in order to deal with the various environmental situations that they face. Since it remains unclear how humans control such interlimb and intralimb coordination during walking, the process has attracted the attention of numerous researchers.

To investigate the underlying mechanism of the interlimb and intralimb coordination in human and animal locomotion, split-belt treadmills have often been used [1–11]. Such treadmills have two parallel belts, whose speeds are controlled independently and are thus capable of artificially creating left–right symmetric and asymmetric environments for examining walking under tied configuration (both belts at same speed) and split-belt configuration (belts travel at different speed) conditions. Under tied configuration (baseline) conditions, the left and right legs move in anti-phase and have similar motions, much as is commonly observed during over-ground walking. However, soon after changing to the split-belt configuration, characteristic locomotion parameters, such as the relative phase between the legs, the duty factor and the centre of pressure (COP) profile, change rapidly. This rapid change is called early adaptation. Moreover, as walking continues using this two-speed belt condition, locomotion parameters related to interlimb coordination, such as the relative phase and COP profile, gradually change and show a behaviour trend towards that coinciding with the baseline state, whereas locomotion parameters related to the intralimb coordination, such as the duty factor, do not show further adaptation. This gradual change in the interlimb coordination is called late adaptation. After late adaptation, the belt speed condition is returned to the tied configuration. This induces a series of rapid changes in the locomotion parameters, including after-effects, which is called early post-adaptation. Then, the varying locomotion parameters related to interlimb coordination gradually return to the baseline state. This process is called late post-adaptation.

**Figure 1.**
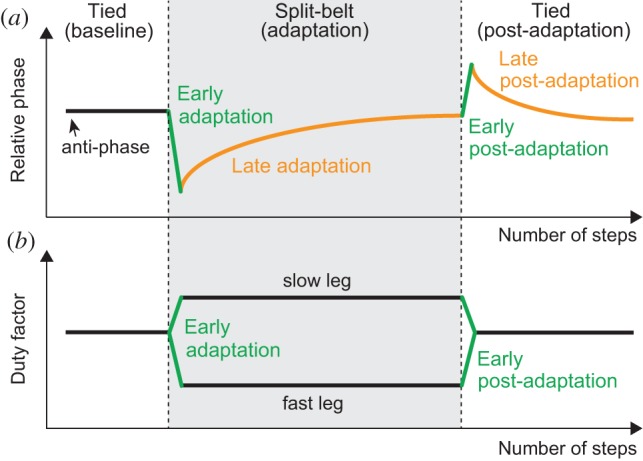
(*a*) Changes in relative phase between legs and (*b*) duty factor in human split-belt treadmill walking (adapted from [8]). When belt speed conditions change from tied to split-belt configuration, these values rapidly change (early adaptation). More specifically, relative phase shifts from anti-phase and the duty factor of the fast leg decreases as that of the slow leg increases. After a period of continuous walking in that condition, the relative phase gradually returns to anti-phase (late adaptation), even though the duty factors remain steady. When belt speed conditions return to the tied configuration, the relative phase rapidly shifts from anti-phase in the opposite direction to the early adaptation, and the duty factors return to the baseline state (early post-adaptation). After a period of continuous walking, the relative phase gradually returns to anti-phase (late post-adaptation).

**Figure 2.**
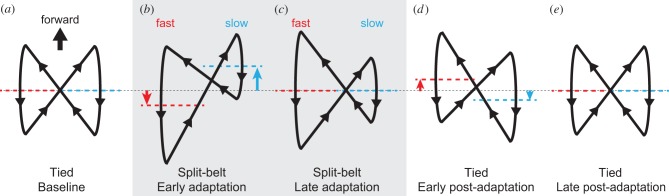
Change in butterfly pattern of COP profile in human split-belt treadmill walking (adapted from [5]). The dotted lines show the centre of each butterfly wing. In (*a*), the tied configuration (baseline), the butterfly wings and centres are almost identical between legs. In (*b*), the early stage of the split-belt configuration (early adaptation), the butterfly wing of the slow leg moves forward, whereas that of fast leg moves backward. In (*c*), the late stage of the split-belt configuration (late adaptation), the butterfly wing centres return to their original positions. In (*d*), the early stage of post-adaptation, the butterfly wings move in the opposite direction to the early adaptation. In (*e*), the late stage of post-adaptation, the butterfly wings return to the baseline state.

More specifically, the relative phase rapidly changes from anti-phase during early adaptation and gradually returns to anti-phase again during late adaptation (figure 1*a*) [8]. During early post-adaptation, the relative phase rapidly shifts in the opposite direction from anti-phase, even when during the tied configuration, which shows after-effects. However, it gradually returns to anti-phase during late post-adaptation. The duty factor of the fast (slow) leg rapidly decreases (increases) during early adaptation, but does not show further change during split-belt configuration walking (figure 1*b*). During early post-adaptation, it rapidly returns to match the baseline state and does not show further change. The COP profile shows a butterfly pattern for one gait cycle, the wings of which are almost symmetrical during the first tied configuration (baseline) walking (figure 2*a*) [5]. As can be seen in the figure, during early adaptation, the wing of the fast (slow) side rapidly moves backward (forward) (figure 2*b*). By contrast, during late adaptation, the wings gradually move so that their centre positions return to their original locations (figure 2*c*). During early post-adaptation, the wings rapidly move in the opposite direction from the early adaptation (figure 2*d*), which shows after-effects, and then gradually return to the baseline state during late post-adaptation (figure 2*e*).

Rapid changes in the locomotion parameters have been observed during split-belt treadmill walking of spinal cats [3,12], which suggests that the early-type adaptations are induced by sensorimotor integration in the spinal cord. On the other hand, since humans with cerebellar damage do not show late adaptation or after-effects during split-belt treadmill walking, it appears that the cerebellum contributes to late-type adaptations and after-effects [6], even though it remains unclear how information processing in these nervous systems induces such adaptations.

When used to identify the contributions of neural information processing to walking adaptation, analytical approaches using measured data and human observation face limitations. To overcome these limitations, attention is being paid to constructive approaches using physical models and robots. In particular, neuro-mechanical models that integrate neural control and mechanical body models have been used to examine physiological hypotheses related to motor control during walking [13–19]. In our previous work [20], we developed a simple spinal cord locomotion control model for use as a walking neural control model based on the physiological concept of a central pattern generator (CPG) and sensory reflexes related to foot contact. We also performed body mechanical model experiments using a bipedal robot walking on a split-belt treadmill. The results obtained via the previous model showed that the robot established stable walking during both the tied and split-belt configurations without requiring changes of the control strategy and parameters. Instead, the relative phase between the legs shifted from anti-phase, and the duty factors changed depending on the speed discrepancy between the belts, which is similar to early adaptation observed in humans. These adaptive behaviours were not the result of specifically designed features in our control model, but occurred because leg motion phases were automatically modulated by immediate responses to the foot contact timing changes necessitated by the speed discrepancy between the left and right treadmill belts. However, because the previous control model did not include a function to regulate motor commands by the cerebellum, gradual locomotion parameter changes, such as late adaptation, and after-effects were not observed, and our model could not fully explain the adaptations observed in human split-belt treadmill walking.

It has been suggested that the cerebellum predicts the sensory consequences of movement based on the efference copy and modifies motor commands through learning based on error information discerned between predicted and actual sensory information [21,22]. In experiments involving encounters with an unexpected hole while walking on a surface, it was shown that the absence of a sensory foot contact afferent at the appropriate time triggers a behaviour-like reflexive reaction [23,24], which suggests that foot contact events are predicted during walking. Furthermore, during split-belt treadmill walking experiments, it was shown that left and right foot contact timings actually change depending on the speed conditions of the treadmill belts [25]. In this paper, we incorporate a cerebellar model to our spinal locomotion control model, which modulates the foot contact timing of each leg via learning, using only the local sensory foot contact information of each leg. We also conducted computer simulations and experiments involving a bipedal robot walking on a split-belt treadmill. Our results show that even though there is no direct control of interlimb coordination, gradual interlimb coordination changes and after-effects appear that are similar to the late adaptation and late post-adaptation changes and after-effects observed in humans. These robot-related adaptation results were then evaluated by comparing them to human split-belt treadmill walking, and the adaptation mechanism was clarified from a dynamic viewpoint.

## 2. Material and methods

### 2.1. Robot experiment mechanical set-up

#### 2.1.1. Biped robot

**Figure 3.**
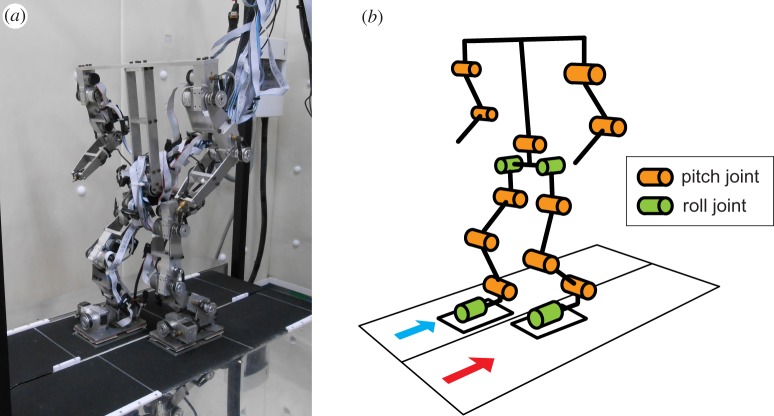
Experimental set-up. (*a*) Bipedal robot and split-belt treadmill and (*b*) schematic model.

In this paper, we used a biped robot (figure 3) developed in our previous work [26]. This robot consists of a trunk composed of two parts, a pair of arms composed of two links, and a pair of legs composed of five links. Each link is connected to the others through a rotational joint with a single degree of freedom. The robot's hip has pitch and roll joints, the knee has a pitch joint, and each ankle has pitch and roll joints. An encoder-equipped motor manipulates each joint. Four touch sensors are attached to the corners of the sole of each foot. The left and right legs are denoted as Legs 1 and 2, respectively. The robot is controlled by an external host computer (Intel Core i5, real-time embedded Linux Xenomai) with 0.2 ms intervals and both computer control and electric power are provided via external cables. During the experiments, the computer control and electric power cables were kept slack and suspended above the walking surface in order to avoid influencing the robot's locomotor behaviour.

The physical model used in our computer simulations was based on the configuration and physical parameters of our robot. To simulate the locomotor behaviour of the robot model, we derived the equations of motion using Lagrangian equations, as in [26,27], and performed forward dynamic simulations by solving the equations of motion using a fourth-order Runge–Kutta method with a step size of 0.1 ms.

#### 2.1.2. Split-belt treadmill for the robot

For the robot experiment, we used the split-belt treadmill (figure 3) developed in our previous work [20], which is equipped with two parallel belts, each of which is equipped with a motor and an encoder to control individual belt speed. The width of each belt is 15 cm and the distance between their rotation axes is 64 cm.

To simulate the robot model walking on a split-belt treadmill, we used two separate floors that move parallel and independently. The foot contact was modelled with the floor using vertical viscoelastic elements and horizontal viscous elements.

### 2.2. Biologically inspired spinal and cerebellar locomotion control models

**Figure 4.**
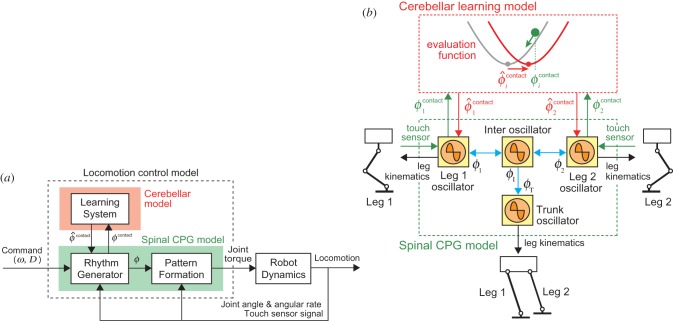
Locomotion control model. (*a*) Two-layered network model composed of spinal CPG and cerebellar learning models. The spinal CPG model consists of the RG and PF models, while the cerebellar learning model receives foot contact timing phase information from the RG model and sends desired (predicted) foot contact timing information to the RG model. (*b*) Phase oscillators in the spinal CPG model and learning in the cerebellar model. Blue arrows indicate interactions between oscillators. Oscillator phases are modulated by phase resetting based on touch sensor signals (green arrows) and desired (predicted) foot contact timing (red arrows). The oscillator phases determine leg kinematics (black arrows). The cerebellar learning model receives phase information at foot contact (green arrows) and modifies the desired (predicted) foot contact timing using the evaluation function, which is sent to the spinal CPG model (red arrows).

We developed a locomotion control model composed of two layers (figure 4); a spinal model that produces motor commands to manipulate the robot based on CPG and sensory reflex, and a cerebellum model that modulates motor commands through learning.

#### 2.2.1. Spinal central pattern generator model

The spinal CPG model developed in our previous works [20,26,28,29] is designed to emulate the sensorimotor properties in the spinal CPG in order to produce adaptive legged robot locomotion. To show the relationship between our spinal CPG and cerebellar learning models, we will briefly explain the spinal CPG model (for details, see [20,26,28,29]).

The spinal CPG model can be visualized as a two-layered hierarchical network composed of the rhythm generator (RG) and the pattern formation (PF) networks [30,31]. The RG network first creates the basic rhythm, and then alters it by producing phase shifts and by performing rhythm resetting in response to sensory afferents (phase resetting). The PF network shapes the rhythm into spatio-temporal motor command patterns. Based on this physiological finding, we developed the spinal CPG model using the following RG and PF models.

For the RG model, we used four simple phase oscillators (Leg 1, Leg 2, trunk and inter oscillators), whose phases are denoted by *ϕ*_1_, *ϕ*_2_, *ϕ*_T_ and *ϕ*_I_. The oscillator phases follow the dynamics2.1
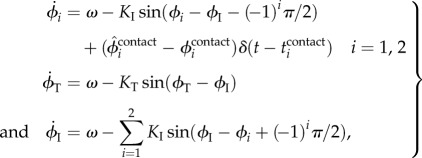
where *ω* is the basic oscillator frequency, 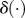 is the Dirac delta function, and *K*_I_ and *K*_T_ are gain parameters. The second terms of the right-hand side of each equation represent the interactions among oscillators necessary to move the relative phase between the leg oscillators into anti-phase. Note that we used a small value for *K*_I_ so that the relative phase can be shifted from anti-phase by phase resetting and learning through locomotion dynamics. The third term of the right-hand side of the equation for the leg oscillators represents phase resetting. Taking inspiration from spinal cats walking on a treadmill, which show how foot contact information influences the locomotion phase and rhythm generated by the CPG [32], we modulated the oscillator phase so that it responds to touch sensor signals based on phase resetting. More specifically, when the foot contact of Leg *i* (*i* = 1, 2) occurs at time 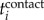 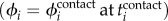, the phase of the Leg *i* oscillator *ϕ_i_* is reset from 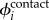 to 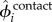. This 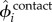 corresponds to the desired (predicted) foot contact timing, as explained in §2.2.2.

**Figure 5.**
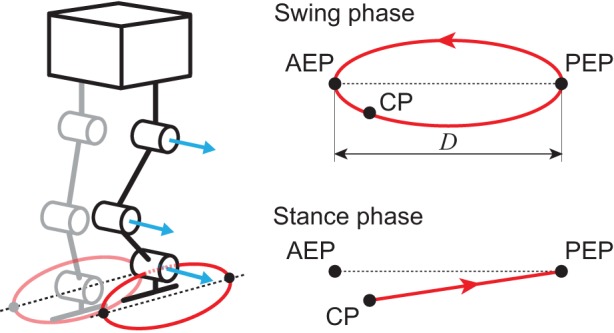
Desired leg kinematics composed of swing and stance phases. At the CP, the trajectory changes from the swing to stance phase. When the ankle pitch joint reaches the PEP, the trajectory moves into the swing phase. The AEP is the desired position at foot contact.

For the PF model, taking inspiration from the physiological finding that spinocerebellar neurons encode the global information of limb kinematics, such as the length and orientation of the limb axis [33–35], we produced the motor commands needed to achieve the desired leg kinematics of the robot based on the oscillator phases obtained from the RG model. We used simple leg kinematics in reference to the length and orientation of the limb axis in the pitch plane, which consists of the swing and stance phases (figure 5). The swing phase is a simple closed curve of the ankle pitch joint that includes an anterior extreme position (AEP) and a posterior extreme position (PEP). It starts from the PEP and continues until the foot makes contact. The AEP corresponds to the desired position at foot contact. The stance phase is a straight line from the contact position (CP) to the PEP. The trajectories for the swing and stance phases are given as functions of the corresponding oscillator phase, where *ϕ_i_* = 0 at the PEP and 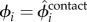 at the AEP (detailed formulation is given in [29]). We denote *D* as the distance between the AEP and PEP, and *T* as the gait cycle (*ω* = 2*π*/*T*). The desired duty factor 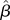, stride length 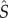 and locomotion speed 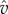 are then given by2.2
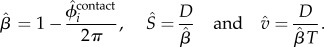

To increase the locomotion stability in three-dimensional space, we also used the hip and ankle roll joints to produce the robot motion in the frontal plane using simple sinusoidal functions based on the trunk oscillator. Because this study focused on the adaptive behaviour of the leg motions on a split-belt treadmill walking, we did not use waist and arm movements. To generate the desired kinematics, each joint is controlled by joint torque based on PD feedback control.

#### 2.2.2. Cerebellar learning model

The cerebellum, which plays an important role in motor control, receives efference copies of motor commands and sensory afferents and then modifies motor commands based on this information [21,22]. It then predicts the sensory consequences of the movement based on the efference copy and determines whether the motor commands are appropriate based on error information differences between the predicted and actual sensory information. The cerebellum continuously modifies motor commands through learning in order to reduce errors.

It has been suggested that the cerebellum predicts the timing of sensory events [36,37] and contributes to achieving tasks that require accurate temporal control [38–40]. Moreover, it has been reported that cerebellum damage impairs motor learning temporal accuracy, although not spatial accuracy [41]. The results of experiments involving walking on a surface with an unexpected hole have shown that the absence of a foot contact sensory afferent at its appropriate (prediction based) timing triggers reflexive-like reaction behaviour [23,24], which suggests that the prediction of foot contact timing is important for motor learning in walking. Furthermore, during split-belt treadmill walking, it was found that foot contact timing actually changes depending on treadmill speed conditions [25]. This, in turn, suggests the importance of foot contact timing prediction and modulation.

In this study, we focus on foot contact timing for the cerebellar learning model. In particular, we modulate desired (predicted) foot contact timing 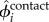 via learning based on the error between the predicted and actual foot contact timings. To accomplish this, we define an evaluation function *V_i,n_* for the *n*th step of Leg *i* using the error between the desired (predicted) foot contact phase 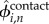 and actual foot contact phase 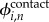 for the *n*th step of Leg *i*, which is given by2.3
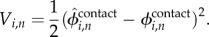


Based on this evaluation function, we then predict the next foot contact time. More specifically, from the gradient direction of the evaluation function, 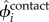 is modulated by2.4
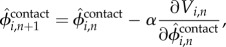
where *α* is the learning rate. Because 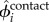 corresponds to the desired timing of the corresponding leg to switch from the swing to the stance phase, this temporal modulation changes the desired duty factor of the corresponding leg (2). Therefore, if a foot contact arrives earlier than predicted, the robot increases the swing leg speed during the next step. In addition, the CP gravitates to alignment with the AEP (figure 5) through this learning.

### 2.3. Robot experiment

For the robot and simulation experiments, we used the following control parameters: *D* = 2.5 cm, *T* = 0.6 s, *K*_I_ = 1.0, *K*_T_ = 10 and *α* = 0.35. For the initial value of 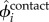, we used *π*, which gives 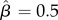, 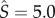 cm and 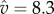 cm s^−1^. The same control parameters were used irrespective of the treadmill speed condition.

For the split-belt treadmill, we denote the speed of the left belt by *v*_1_ and that of the right belt by *v*_2_. At the beginning, the robot walked with the treadmill in the tied configuration using *v*_1_ = *v*_2_ = 7.9 cm s^−1^ (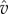 was set to be slightly larger than *v*_1_ and *v*_2_ so that the robot remained centre of the treadmill, because 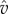 is the desired locomotion speed defined by the desired duty factor and gait cycle in (2.2) and is not necessarily achieved). After the robot established a steady gait, we suddenly changed the speed condition from tied to split-belt configuration using *v*_1_ = 9.7 and *v*_2_ = 6.1 cm s^−1^. After the robot walked in the split-belt configuration for a sufficient amount of time, we suddenly returned the speed condition to the tied configuration. We performed this robot trial experiment six times and investigated the robot's behaviour from the averages of the results obtained before and after the belt condition changed from the tied to split-belt configuration and the results before and after the belt condition changed from the split-belt back to the tied configuration. This was necessary because different trials have different numbers of steps for each period.

### 2.4. Measurement of human split-belt treadmill walking

To evaluate the biological relevance of our findings from the robot and simulation experiments, we measured human walking behaviour on a split-belt treadmill (ITR3017, Bertec Corporation) that was equipped with two separate belts and an embedded force plate underneath each belt. The participants, who were five healthy men (ages: 22–24, weights: 51–74 kg, and heights: 163–170 cm), were instructed to hold onto the bar installed at the front of the treadmill and wore a safety harness with cords that were slack and suspended above the treadmill during the experiment to ensure that they did not affect the walker's locomotor behaviour.

**Figure 6.**
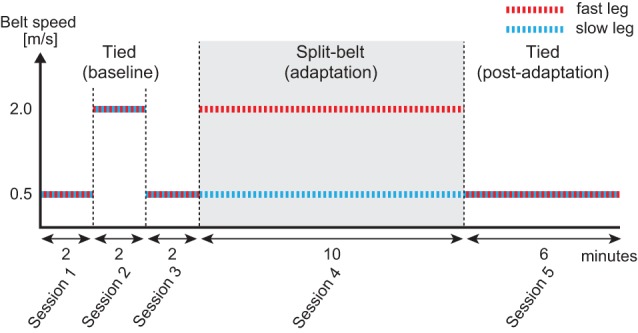
Protocol for human split-belt treadmill walking experiment composed of five sessions. Session 1 is a slow tied configuration for 2 min, Session 2 is a fast tied configuration for 2 min, Session 3 is a slow tied configuration for 2 min, Session 4 is the split-belt configuration for 10 min and Session 5 is a slow tied configuration for 6 min.

Each trial consists of five sessions based on the previous work [8] (figure 6) and each participant conducted one trial. In Session 1, the participants walked with the tied configuration using *v*_1_ = *v*_2_ *=* 0.5 m s^−1^ for 2 min. In Sessions 2 and 3, they again walked for 2 min with the tied configuration using *v*_1_ = *v*_2_ = 2.0 m s^−1^ and *v*_1_ = *v*_2_ *=* 0.5 m s^−1^, respectively. In Session 4, they walked for 10 min with the split-belt configuration using *v*_1_ = 0.5 and *v*_2_ = 2.0 m s^−1^. In Session 5, they walked for 6 min with the tied configuration, again using *v*_1_ = *v*_2_ = 0.5 m s^−1^. The time interval between sessions was, at most, 1 min, which was just long enough to change the treadmill speed condition.

Kinematics were measured with a motion capture system (Mac 3D Digital RealTime System, Motion Analysis Corporation). The motion capture and force plate sampling rates were set at 500 Hz. Reflective markers were attached to the participants at the following locations: the head and both hemibodies of upper limit of the acromion, elbow, wrist, greater trochanter, lateral condyle of the knee, lateral malleolus, second metatarsal head, and heel. The measured kinematic and force data were low-pass filtered at 6 Hz (with a second-order Butterworth filter). The centre of mass (COM) was computed using the kinematic data, while the COP was calculated using the force and kinematic data. To see the COP relative to the body, we projected the COM on the ground and computed COP–COM.

As shown by Mawase *et al.* [5], the COP profile changes during human split-belt treadmill walking (figure 2). This change reflects the stride and step length changes shown by Reisman *et al.* [8], because the stride length is related to the vertical length of the COP butterfly wing, while the step length is related to the relative position of both wings. The remarkable point here is the way in which the centre positions of the butterfly wings change. More specifically, while the centre positions of both legs are almost the same during tied configuration (baseline) walking, soon after the split-belt configuration starts, the wing on the slow side moves forward, whereas the wing on the fast side moves backward (early adaptation). This induces differences between their centre positions. However, after a while, the wings return to their original positions and their centre positions nearly coincide again (late adaptation). Furthermore, when the speed condition is returned to the tied configuration, the wing on the slow side moves backward, the wing on the fast side moves forward and their centre positions differ again (early post-adaptation). However, their moving directions are opposite to those in the early adaptation, which shows after-effects. After a while, their centre positions gradually return to the baseline state and the difference disappears (late post-adaptation). That is, the relative positions of their centres change depending on the configuration and stage of the treadmill speed condition.

In this paper, to clearly show this change, we investigated the left–right difference of the centres of the butterfly wings of the COP pattern. For statistical analysis, we used averages of the first five steps in Session 1 for the baseline state, the first and last five steps in Session 4 for the early and late adaptation stages, and the first and last five steps in Session 5 for the early and late post-adaptation stages. In this process, the measured COP data of each participant were obtained by normalizing using the mean stride length in the tied configuration. We used one-way repeated-measures analysis of variance (ANOVA) to compare the differences between the five testing intervals (baseline period, early and late stages of adaptation periods, and early and late stages of post-adaptation periods). When the ANOVA showed a significant difference, we conducted post hoc analysis using Tukey's honestly significant different test.

## 3. Results

### 3.1. Relative phase between legs

**Figure 7.**
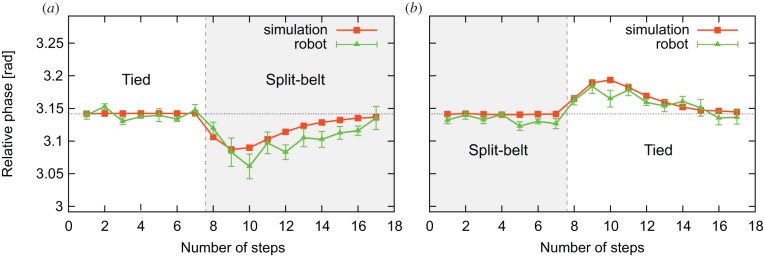
Relative phase between leg oscillators for simulation and robot experiments. Panels (*a*) and (*b*) show the results for the adaptation and post-adaptation periods, respectively. For the robot experiment, data points and error bars are the means and standard error results of six experiments. During the first tied configuration, the relative phase is anti-phase. After changing to the split-belt configuration, it rapidly shifts downward, and then gradually returns to anti-phase. After returning to the tied configuration, it rapidly shifts upward, and then gradually returns to anti-phase.

Figure 7*a*,*b* shows the relative phase between the leg oscillators, which corresponds to the relative phase between the legs, for the computer simulation and robot experiment using the average value for one gait cycle by 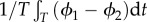 for the adaptation and post-adaptation periods, respectively. For the robot experiment, the data points and error bars are the means and standard error results of six experiments. As can be seen in figure 7*a*, the relative phase shows anti-phase during the first tied configuration. However, it rapidly shifts downward from anti-phase soon after the switchover to the split-belt configuration, and then gradually returns to anti-phase. As shown in figure 7*b*, it rapidly shifts upward from anti-phase soon after the return to the tied configuration, and then gradually returns to anti-phase.

**Figure 8.**
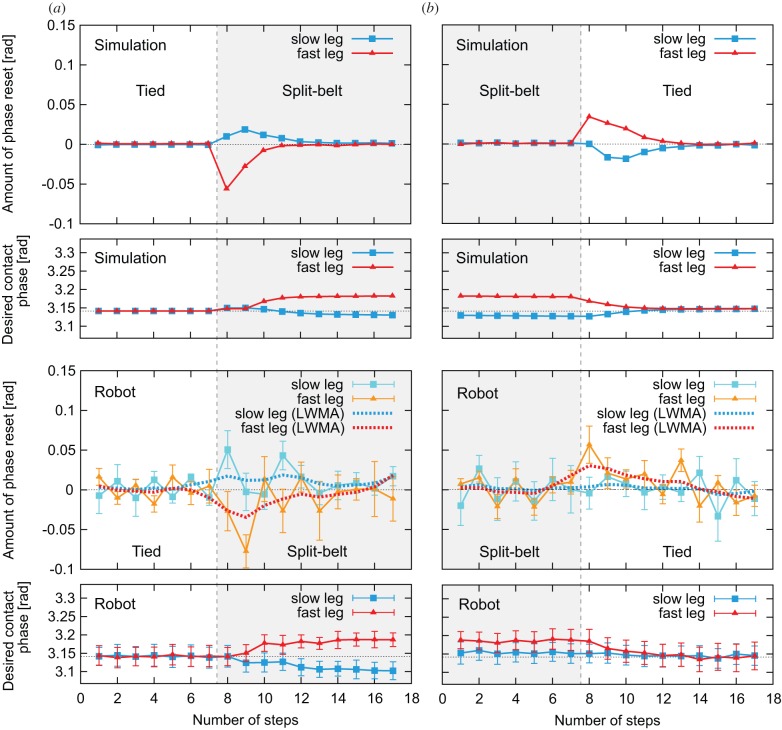
Amount of phase resetting and desired foot contact phase of leg oscillators for simulation and robot experiments. Panels (*a*) and (*b*) show the results for adaptation and post-adaptation periods, respectively. For the robot experiment, data points and error bars are the means and standard error results of six experiments, while the dotted lines are the five-period LWMA. Here, the resetting amount is zero before the belt condition change, but appears soon after the belt condition change. This induces the modulation of desired foot contact phases. After a while, the resetting amount gradually returns to zero and desired foot contact phases converge.

Figure 8*a*,*b* shows the amount of phase resetting 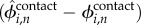 at a foot contact whose square value corresponds to the evaluation function *V_i_*_,*n*_ for learning, and the desired foot contact phase 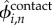 for the adaptation and post-adaptation periods, respectively. When the amount of phase resetting is positive (negative), the foot contact occurs earlier (later) than the predicted timing. As can be seen in figure 8*a*, this amount is almost zero at the first tied configuration, but appears soon after the split-belt configuration starts, which induces the modulation of the desired foot contact phase of each leg. After a while, the resetting amount returns to zero and the desired foot contact phases converge, thus indicating that learning is complete. However, when the belt condition returns to the tied configuration, the amount of phase resetting appears again, which changes the desired foot contact phases, as shown in figure 8*b*. After a while, the resetting amount vanishes, the desired foot contact phases return to the original values, and the learning is again complete. Although the robot experiments show variations for the phase resetting amount, the moving average (five-period linear weighted moving average (LWMA)) clearly shows these properties.

It can be seen that the results of the robot and simulation experiments are qualitatively and quantitatively similar. Moreover, the relative phase has a qualitatively similar trend to the early and late stages of adaptation and post-adaptation periods observed during human split-belt treadmill walking (figure 1*a*).

### 3.2. Duty factors

**Figure 9.**
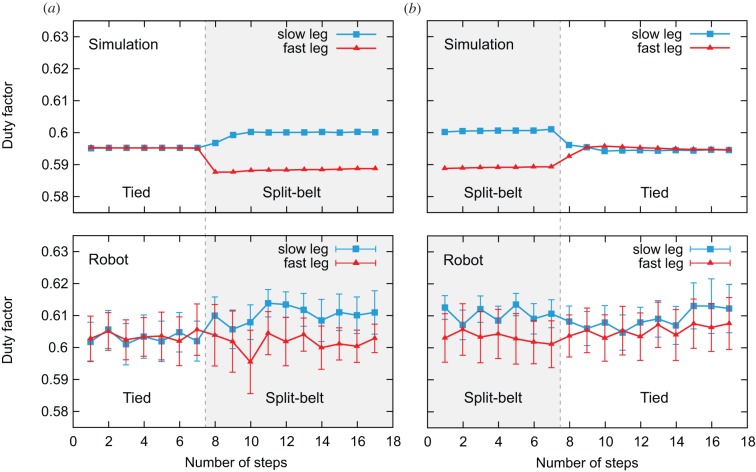
Duty factors of legs for simulation and robot experiments. Panels (*a*) and (*b*) show the results for adaptation and post-adaptation periods, respectively. For the robot experiment, data points and error bars are the means and standard error results of six experiments. The duty factors are identical between legs during the first tied configuration, change rapidly soon after the split-belt configuration starts and remain stable after the rapid change. Soon after returning to the tied configuration, they rapidly return to the baseline state, and then stabilize.

Figure 9*a*,*b* shows the adaptation and post-adaptation period duty factors of the legs for the computer simulation and robot experiment. As can be seen in figure 9*a*, during the first tied configuration, the duty factors are identical between legs. However, soon after changing to the split-belt configuration, the duty factor of the fast leg rapidly decreases, while that of the slow leg increases. These new duty factors remain in place after the rapid changes, unlike the relative phase between the legs (figure 7). As shown in figure 9*b*, duty factors rapidly return to the baseline state soon after changing back to the tied configuration, and then stabilize.

Although the robot experiment duty factors were slightly larger than those for the computer simulation, the results of the robot and simulation experiments show similar qualitative and quantitative trends. Furthermore, much like the relative phase of the legs, the duty factor results have a qualitatively similar trend to human split-belt treadmill walking (figure 1*b*).

### 3.3. Centre of pressure

**Figure 10.**
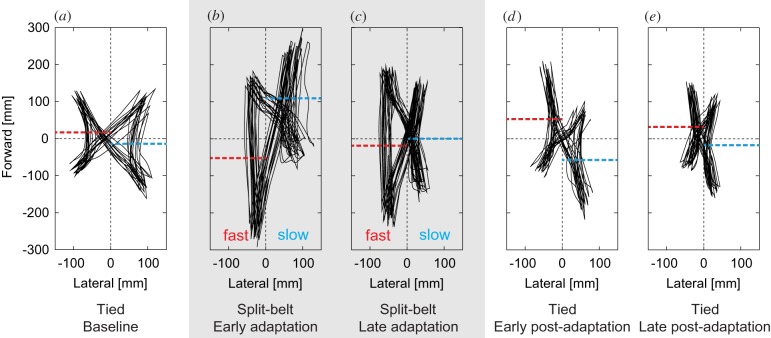
COP profile relative to COM measured during human split-belt treadmill walking for (*a*) the baseline in tied configuration (first 20 s of Session 1), (*b*) the early stage of adaptation in split-belt configuration (first 20 s of Session 4), (*c*) the late stage of adaptation in split-belt configuration (last 20 s of Session 4), (*d*) the early stage of post-adaptation in tied configuration (first 20 s of Session 5) and (*e*) the late stage of post-adaptation in tied configuration (last 20 s of Session 5). Dotted lines show centre average of each butterfly wing.

Figure 10*a*–*e* shows the COP profile of one participant using the first 20 s of data during the tied configuration of Session 1, the first 20 s of data during the split-belt configuration of Session 4, the last 20 s of data of Session 4, the first 20 s of data during the tied configuration of Session 5 and the last 20 s of data of Session 5, respectively. The dotted lines show average centre position of each butterfly wing of the COP pattern. During the first tied configuration, the butterfly wings were almost identical between legs, so their centre positions coincided (figure 10*a*). Soon after the start of the split-belt configuration, the wing of the slow side moved forward, while the wing of the fast side moved backward, which induced differences between their centre positions (figure 10*b*). After a while, they moved so that their centre positions almost coincided again (figure 10*c*). Soon after the return to the tied configuration, the wing of the slow side moved backward, while the wing of the fast side moved forward (figure 10*d*). The directions were opposite to those in figure 10*b* and their centre positions differed again. After a while, their centre positions once again approached the baseline state (figure 10*e*). These features are consistent with the report by Mawase *et al.* [5] (figure 2).

**Figure 11.**
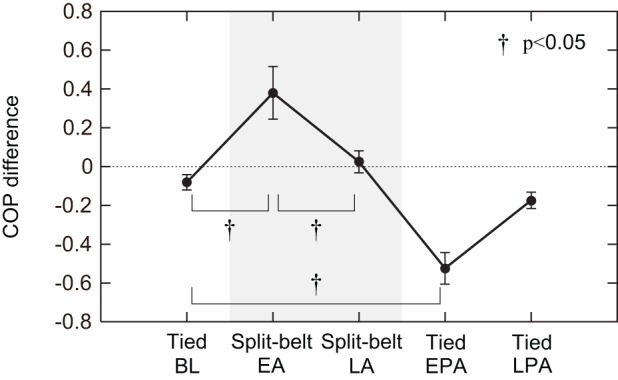
Left–right differences for the butterfly wing centre of COP patterns of the five participants for five intervals (baseline period of tied configuration, early and late adaptation periods of split-belt configuration, and early and late post-adaptation periods of tied configuration). A positive value indicates that the slow side is further forward than the fast side. Data points and error bars are the means and standard errors of the five participants. During the first tied configuration, differences were almost zero (centre position was almost identical between legs). Positive differences appeared in the early stage of the split-belt configuration but declined to almost zero in the late stage of the split-belt configuration. During the early stage of post-adaptation, a negative difference appeared but almost vanished again in the late stage of post-adaptation. BL, baseline; EA, early adaptation; LA, late adaptation; EPA, early post-adaptation; LPA, late post-adaptation.

To clearly show these changes for all participants, we then investigated the left–right butterfly wing centre differences. Figure 11 shows the means and the standard error results of the five participants for the baseline period of the tied configuration, the early and late adaptation periods of the split-belt configuration, and the early and late post-adaptation periods of the tied configuration. When this difference is positive, the slow side is further forward than the fast side. In the first tied configuration, the difference was almost zero, indicating that the centre positions between the legs nearly coincided. The positive difference occurred during the early stage of the split-belt configuration, but declined to nearly zero again during the late stage of the split-belt configuration. During the early stage of post-adaptation, the negative difference appeared, but almost vanished again during the late stage of post-adaptation. ANOVA identified a significant main effect for periods (*p* < 0.05), and post hoc testing revealed significant changes in the butterfly wing centre differences between the first tied configuration and the early stage of the split-belt configuration (*p* < 0.05), between the early and late stages of the split-belt configuration (*p* < 0.05), and between the first tied configuration and the early stage of the post-adaptation (*p* < 0.05). However, the differences between the first tied configuration and the late stage of the split-belt configuration and between the first tied configuration and the late stage of the post-adaptation were negligible and no significant differences could be found (*p* = 0.87 and 0.90).

**Figure 12.**
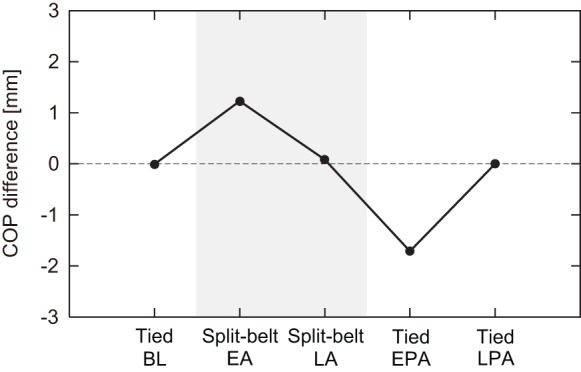
Simulation results of the left–right butterfly wing centre of COP pattern differences for five intervals (baseline period of tied configuration, early and late adaptation periods of split-belt configuration, and early and late post-adaptation periods of tied configuration). Positive values indicate that the slow side is further forward than the fast side. During the first tied configuration, differences were zero (centre position was identical between legs). Positive differences occurred in the early stage of the split-belt configuration, but declined to almost zero in the late stage of the split-belt configuration. Negative differences appeared in the early post-adaptation period, but vanished again in the late post-adaptation period. BL, baseline; EA, early adaptation; LA, late adaptation; EPA, early post-adaptation; LPA, late post-adaptation.

Figure 12 shows the computer simulation results for the difference centre of the COP pattern butterfly wings. Note that due to the lack of a force plate in the split-belt treadmill that would allow the COP to be calculated for the robot, there are no data for the robot experiments. However, the robot experiments are expected to have similar properties for the COP results recorded in the simulation, as shown in figures 7–9. As can be seen in the figures, there were no differences between the legs during the first tied configuration, but positive differences appeared at the early stage of the split-belt configuration that decreased to almost zero by the late stage of the split-belt configuration. Although the return to the tied configuration induced a negative difference, it declined to zero again in the late stage of post-adaptation. These trends are qualitatively similar to those observed in the human split-belt treadmill walking experiments (figure 11).

## 4. Discussion

In this paper, we report on the development of spinal cord and cerebellum control walking models based on physiological findings. For the spinal model, we determined motor commands using an oscillator network based on the CPG while incorporating sensory reflexes based on foot contact information. For the cerebellar model, we modified the motor commands based on error information differences between the predicted and actual foot contact timings obtained through learning. We then performed robot and simulation experiments involving a bipedal robot walking on a split-belt treadmill to investigate what forms of adaptation appear and what mechanisms explain such adaptations. Our results show that characteristic locomotion parameters, such as the relative phase between the legs and their duty factors, exhibit early and late adaptation and early and late post-adaptation trends that are similar to those observed in human split-belt treadmill walking.

### 4.1. Adaptation mechanism from a dynamic viewpoint

As Reisman *et al.* [8] pointed out, only the locomotion parameters involved in the interlimb pattern change gradually in the late adaptation and late post-adaptation periods and show after-effects during human split-belt treadmill walking. Our adaptation results were induced by sensory reflexes and learning based solely on local foot contact information for each leg. Despite our model's lack of direct interlimb coordination control, the early- and late-type adaptations and after-effects that were observed in interlimb coordination showed strong similarities to those observed in humans. We will discuss this adaptation mechanism from a dynamic viewpoint below.

**Figure 13.**
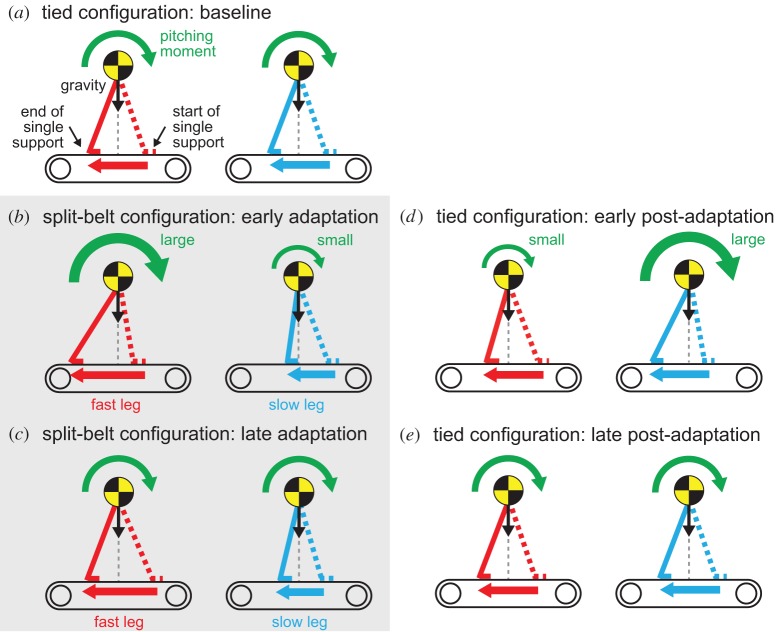
Pitching moment change due to belt speed changes. In (*a*), the baseline period, the pitching moment is identical between legs. In (*b*), the early adaptation period, the fast side belt speed acceleration increases the pitching moment during the single support phase of the fast leg, which induces early contralateral (slow) leg foot contact. A similar mechanism is also applied to the other side and delays fast leg foot contact. In (*c*), the late adaptation period, the difference of pitching moment between the legs is reduced by moving the position of the fast side support leg forward relative to the COM, and moving that of the slow side backward. In (*d*), the early post-adaptation period, the fast side belt speed deceleration caused by the return to the tied configuration decreases the pitching moment, which induces late contralateral (slow) leg foot contact. In (*e*), the late post-adaptation period, the pitching moment difference is reduced by moving the position of the fast side support leg backward and moving that of the slow side forward.

As reported by MacLellan *et al.* [25], foot contact timing of the slow (fast) leg becomes earlier (later) when the speed condition changes from the tied to the split-belt configuration. This change is induced by the pitching moment changes of the body in the sagittal plane that results from the speed discrepancy between the belts. More specifically, during the tied configuration, the pitching moments between the legs are identical (figure 13*a*). However, soon after the split-belt configuration starts, the fast leg pitching moment increases during the single support phase due to the belt speed increase, which pulls the fast leg (figure 13*b*). This, in turn, induces early foot contact of the contralateral (slow) leg. A similar mechanism is applied to the other side, resulting in delayed fast leg foot contact. These foot contact timing changes could be verified from the amount of phase resetting in our model (figure 8) and were found to have induced the relative phase shifts between the legs from anti-phase in the early adaptation stage (figure 7).

During late adaptation, the pitching moment difference between the legs declines due to the gradual modulation of foot contact timing achieved through learning, which permits the relative phase to return to anti-phase. This occurs because the position of the support leg relative to the COM changes to reduce the difference. More specifically, the position of the support leg on the fast side moves forward relative to the COM, which decreases the pitching moment induced by gravity, while the position of the support leg of the slow side moves backward, which increases the gravity-induced pitching moment (figure 13*c*). In the next paragraph, we will explain the reason why the positions of the support leg move relative to the COM due to the modification of the foot contact timing. Because the vertical lines of the butterfly wings in the COP profile show the support position of the legs during the single support phase, these changes in the support position can be verified from figure 11 for humans and from figure 12 for our model.

**Figure 14.**
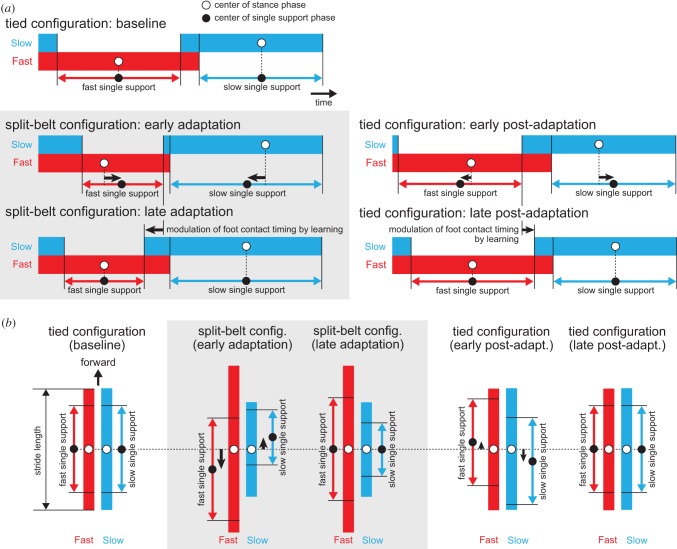
Change in (*a*) temporal and (*b*) spatial relationships between stance and single support phases of each leg for each configuration and stage of the speed condition. The white and black circles indicate stance and single support phase centres, respectively. In the first tied configuration, these centres coincide in each leg. In the early stage of the split-belt configuration, the centres differ. In the late stage of the split-belt configuration, their centres coincide once again. In the early post-adaptation period, the centres become different in the opposite direction to the early adaptation period. In the late post-adaptation period, their centres coincide once again.

Next, we will explain the reason why the positions of the support leg move relative to the COM due to the foot contact timing modification. Figure 14*a*,*b* shows the temporal and spatial relationships, respectively, between the stance phase and single support phase durations of each leg for each configuration and stage of the speed condition. During the first tied configuration, the timing and position of the stance phase centre (white circles) are identical to those of the single support phase (black circles) in each leg. In the early stage of the split-belt configuration, the stance and single support phase centres for both the timing and position become different due to the changes in the relative phase between the legs and duty factors. More specifically, in the fast (slow) leg, the timing of the single support phase centre comes later (earlier) than that of the stance phase. Similarly, in the fast (slow) leg, the position of the single support phase centre moves further backward (forward) than that of the stance phase. During the late stage of the split-belt configuration, learning modulates the movement in order to reduce the difference between the predicted and actual foot contact timings. In the slow leg, because the actual foot contact timing was earlier than predicted at the early stage of the split-belt configuration, the predicted timing becomes earlier through learning, which then increases the swing movement speed. As a result, actual foot contact timing at the late stage of the split-belt configuration comes earlier than that in the early stage, just as is observed in humans [8,25]. This reduces the timing difference between the stance phase and single support phase centres in each leg. This timing modulation shows that, in the fast (slow) leg, the single support phase position moves forward (backward) relative to that of the stance phase, and that their centre positions in each leg once again coincide.

This mechanism is also applied to the post-adaptation period. Adaptive behaviours and after-effects appear during this period because the belt speed condition returns to the tied configuration after learning the foot contact timing in each leg in order to adapt to the split-belt configuration. However, because the acceleration and deceleration in the belt speed change are different, the changing trends of locomotion parameters and learning occur in the opposite direction to that of the adaptation period, as illustrated in figures 13*d* and 14*a*,*b*. After a period of continuous walking, physical conditions return to the baseline state, as illustrated in figures 13*e* and 14*a*,*b*.

It has been suggested that controlling the COM position contributes to improving locomotion stability during the late-type adaptations of human split-belt treadmill walking [4,5,8], which supports the adaptation mechanism discussed above. However, note that the early- and late-type adaptation results of our robot experiments were not characteristics that we specifically designed into our control model. Instead, they emerged through the dynamic interactions occurring between the robot mechanical system, the spinal- and cerebellar-based locomotion control system, and the environment.

### 4.2. Contributions of spinal cord and cerebellum to locomotor adaptation

Adaptation in human split-belt treadmill walking can be classified using two different timescales. These are early- and late-type adaptations, and they are primarily produced by the contributions of different layers in the neural system: the spinal cord and the cerebellum. The spinal cord produces motor commands through the RG and PF networks [30,31] and modulates them immediately in response to sensory input [42]. In fact, spinal cats walking on a split-belt treadmill showed rapid adaptive behaviour much like early adaptation [3,12]. Our spinal CPG model [20] (without cerebellar learning) also showed rapid adaptive behaviour much like early adaptation. The cerebellum receives efference copy information from the spinal cord through the ventral spinocerebellar tract and sensory information through the dorsal spinocerebellar tract [43,44]. Purkinje cells produce the output of the cerebellar cortex in order to modulate motor commands based on error information between the sensory information predicted via the efference copy and the actual sensory information. This modification contributes to late-type adaptations, as suggested from the fact that humans with cerebellar damage do not show late adaptation behaviours and after-effects [6]. The reflexive response in the spinal cord and learning modulation in the cerebellum induce these two different adaptation timescales. The reflexive response in the spinal cord secures the ability to continue walking against environmental changes, and the cerebellum modulates the movements under those conditions to make walking smoother and more energy efficient [45]. Our two-layered model, which consists of the spinal CPG model (with reflexive modulation of motor commands based on phase resetting) and the cerebellar model (with gradual modulation of the commands through learning), produced two such different adaptation timescales.

### 4.3. Prediction and learning through evaluation in the cerebellum

In this paper, we modelled a cerebellum function that contributes to coordinated movements through predictions in order to investigate late-type adaptations and after-effects in human split-belt treadmill walking. For example, when moving an arm while standing, humans modulate their posture before the arm movement in order to maintain the stability against the COM perturbation caused by the arm movement itself [46]. The cerebellum contributes to this anticipatory regulation. During locomotion, phase modulation responding to the stimulation of nerves in the legs [47–50] and reflexive reaction in the absence of foot contact sensory information [23,24] suggest that sensory information related to foot contact timing plays an important role in modulating locomotor behaviour. This is especially notable in split-belt treadmill walking, where, soon after the split-belt configuration starts, the vertical ground reaction forces at the foot contact timing (early stance phase) increase rapidly, and then gradually decline [5]. By contrast, no changes are observed during middle and late stance phases in the split-belt configuration. It has been reported that ankle stiffness was predictively modulated at foot contact, which changes the ground reaction forces [51]. Furthermore, climbing fibre responses of cerebellar Purkinje cells, which represent error information for motor control, increased around foot contact [11]. These observations suggest that the environmental change at the early stage of the split-belt configuration induced the difference between the predicted and actual foot contact timings, and thus only increased the ground reaction forces in the early stance phase. Modification of the predicted timing performed in order to adapt to the environmental change was found to reduce the ground reaction forces. We incorporated a learning model to regulate foot contact timing based on error information between predicted and actual foot contact timings, which changed characteristic locomotion parameters, such as the relative phase between the legs, duty factors and COP patterns, much as those observed in human split-belt treadmill walking. Therefore, our modelling and results are consistent with observations in humans and clarify the importance of foot contact timing modification for adaptive locomotion from a dynamic viewpoint.

Humans predict something through the evaluation of various parameters and enhance their movements through learning in motor control. The cerebellum contributes to this prediction and learning. However, since the underlying mechanism remains unclear, modelling studies have been attracting attention. For example, for human arm movements, learning models that aim to minimize jerk or torque-change have been proposed [52,53]. However, for human locomotion, it remains unclear what parameters are predicted and how to facilitate the learning. This is partly because locomotion is a whole-body movement through the limb-movement and posture controls and is governed by complicated dynamics including foot contact and lift off, which change the physical constraints. During human split-belt treadmill walking, various parameters, such as the relative phase between legs, COP patterns, muscle activities and ground reaction forces, gradually change. These are expected to be attributed to the prediction and learning processes. However, the prediction and learning processes of other parameters might also cause these changes. Our relative phase and COP pattern results, which are the results of the prediction and learning of foot contact timing of each leg, provide an example of such a case. Modelling studies are also useful for examining the possibility of potential parameters through the comparison of the results obtained from human measured data and the clarification of dynamical mechanisms.

### 4.4. Controlling the global pattern through local information

In this study, sensory reflexes and learning about foot contact timing for each leg resulted in appropriate modifications to interlimb coordination. This means that the global walking pattern (interlimb coordination) was manipulated through the modification of local information of each leg (foot contact timing) because the left and right legs are connected through the trunk, which means that a foot contact timing modification of one leg affects, and is affected by, the other leg. Therefore, even if modifications are performed separately in each leg, they influence the whole-body movement. In other words, solving a low-order problem using local information can produce appropriate whole-body movement without making it necessary to solve a high-order problem that will determine the whole-body movement using whole-body information. This idea is expected to be useful for control design of legged robots because it will allow adaptive locomotion using a small number of sensors.

### 4.5. Modification of spatio-temporal movement patterns

Humans modulate the spatio-temporal patterns of their movements in order to adapt to environmental changes. Walking on a split-belt treadmill is useful for visualizing the adaptation mechanism in the spatial and temporal patterns. In our model, we focused solely on the temporal pattern, that is, foot contact timing for the learning model. We found that the temporal modification induced not only changes in the locomotion temporal patterns, such as the relative phase between legs, but also changes in the spatial pattern, such as the COP pattern. This means that the temporal modification of the robot movement induced the spatial modification through locomotion dynamics, as explained in §4.1. However, from the human measurements, it is difficult to identify which pattern is manipulated and which induces the modification of the other pattern. Our modelling approach can be used to demonstrate the human gait strategy, which is difficult to clarify from measurements.

### 4.6. Limitations of our approach and future work

In this study, we used a robotic platform to investigate human bipedal walking. The robot mechanical system is much simpler than the human musculoskeletal system. In addition, the robot body is rigid and motors strictly control its joints, whereas humans are more flexible because muscles manipulate their joints. Moreover, we used a much simpler locomotion control model than the human neural locomotion control system. Even though these differences caused quantitative differences in locomotion parameters, it is clear that our robot showed trends in adaptive behaviour that were similar to those of humans in split-belt treadmill walking, as was confirmed by the comparisons with humans. This suggests that our simple robot mechanical and locomotion control systems are capable of capturing the essential aspects needed to generate the adaptive locomotor behaviour observed in humans.

While cerebellar damage causes gait ataxia, the cerebellum has numerous other functions related to adaptive locomotion, in addition to the interlimb and intralimb coordination observed in split-belt treadmill walking. For example, the cerebellum plays a crucial role in the dynamic regulation of balance that is necessary to stabilize walking behaviour [54]. Additionally, it contributes to motor control of voluntary and intentional leg movements, such as stepping over obstacles [55]. To further clarify the cerebellar underlying mechanisms in walking, we intend to develop a more sophisticated model and a biologically plausible robot for use in our future studies.
